# Supplementary material for: Characteristics of the Salivary Microbiota in Periodontal Diseases and Potential Roles of Individual Bacterial Species To Predict the Severity of Periodontal Disease
Source: Microbiol Spectr. 2023 May 16;11(3):e04327-22. doi: 10.1128/spectrum.04327-22 (PMC10269672; doi:10.1128/spectrum.04327-22)
Supplement: Supplemental file 1 — Supplemental material. Download spectrum.04327-22-s0001.pdf, PDF file, 0.7 MB [file spectrum.04327-22-s0001.pdf]

Supplemental 1. Bacterial species-specific primer

SPECIES	GENE	SEQUENCE OF PRIMERS (5’ → 3’)
Universal primer (1)	16S rDNA	F-GTG STG CAY GGY TGT CGT CA<br>R-ACG TCR TCC MCA CCT TCC TC
P. gingivalis (2)	*rpoB*	F-GGA AGA GAA GAC CGT AGC ACA AGG A<br>R-GAG TAG GCG AAA CGT CCA TCA GGT C
T. forsythia (3)	*rpoB*	F-GGATTGACCACCGGCGAAGACA<br>R-CGGACACGACGGTTACTCAAATGG
T. denticola (3)	*rpoB*	F-CGGGCGTGCATCTTGTCGTCTAC<br>R-CTTAACCGGCCGCCTCTTTGAA
P. intermedia (4)	*rpoB*	F-ACC CAT TGG CAG AAG TTA CG<br>R-TCA ATA GGA CAC AAG CGA CCA TAG
P. endodontalis (3) [21]	*rpoB*	F-AGCAGAGTTCCGTCGTCGTATTCA<br>R-GCGCCCTGCCATCTTGTCTC
F. alocis (5)	16S rDNA	F-AACCGGAGCAAAACTGAGAA<br>R-CCGTCCGCCACTAACTTCTA
F. nucleatum (3)	*rpoB*	F-ACCTAAGGGAGAAACAGAACCA<br>R-CCTGCCTTTAATTCATCTCCAT
P. micra (6)	*fusA*	F-AGAATCAATTTCTCAAGGTGCAG<br>R-CAATGTCCATATTGTCCACTACCA
R. dentocariosa (3)	*rpoB*	F-CTGGGCAAAGCGTCTGGAAAAC<br>R-GAAATCACGAATCGGGGAAATCTC

References

1. Maeda H, Fujimoto C, Haruki Y, Maeda T, Kokeguchi S, Petelin M, Arai H, Tanimoto I, Nishimura F, Takashiba S. 2003. Quantitative real-time PCR using TaqMan and SYBR Green for Actinobacillus actinomycetemcomitans, Porphyromonas gingivalis, Prevotella intermedia, tetQ gene and total bacteria. FEMS Immunol Med Microbiol 39:81-86. https://doi: 10.1016/S0928-8244(03)00224-4.

2. Park SN, Park JY, Kook JK. 2011. Development of Porphyromonas gingivalis-specific quantitative real-time PCR primers based on the nucleotide sequence of rpoB. J Microbiol 49:315-319. https://doi.org/10.1007/s12275-011-1028-y.

3. Park SN, Lim YK, Kook JK. 2013. Development of quantitative real-time PCR primers for detecting 42 oral bacterial species. Arch Microbiol 195:473-482. https://doi.org/10.1007/s00203-013-0896-4.

4 Park S-N, Kook J-K. 2015. Development of quantitative real-time PCR primers for detection of Prevotella intermedia. International Journal of Oral Biology 40:205-210.

5 Spooner R, Weigel KM, Harrison PL, Lee K, Cangelosi GA, Yilmaz O. 2016. In Situ Anabolic Activity of Periodontal Pathogens Porphyromonas gingivalis and Filifactor alocis in Chronic Periodontitis. Sci Rep 6:33638. https://doi.org/10.1038/srep33638.

6 Tomas I, Regueira-Iglesias A, Lopez M, Arias-Bujanda N, Novoa L, Balsa-Castro C, Tomas M. 2017. Quantification by qPCR of Pathobionts in Chronic Periodontitis: Development of Predictive Models of Disease Severity at Site-Specific Level. Front Microbiol 8:1443. https://doi.org/10.3389/fmicb.2017.01443.

Supplemental 2.

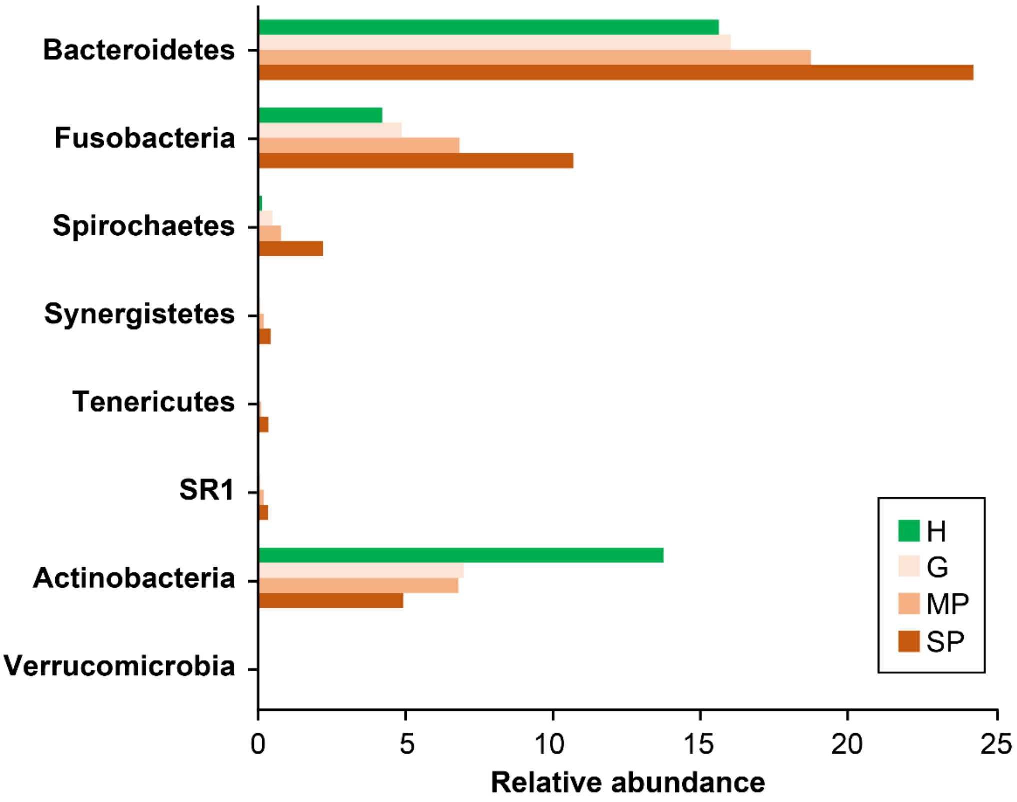

Differences in taxa distribution among four groups at the phylum. Eight phyla significantly different in saliva among four groups *$p < 0.05$ by Kruskal–Wallis H test among groups. $p < 0.05$ was considered statistically significant by Kruskal–Wallis H test.

Supplemental 3.

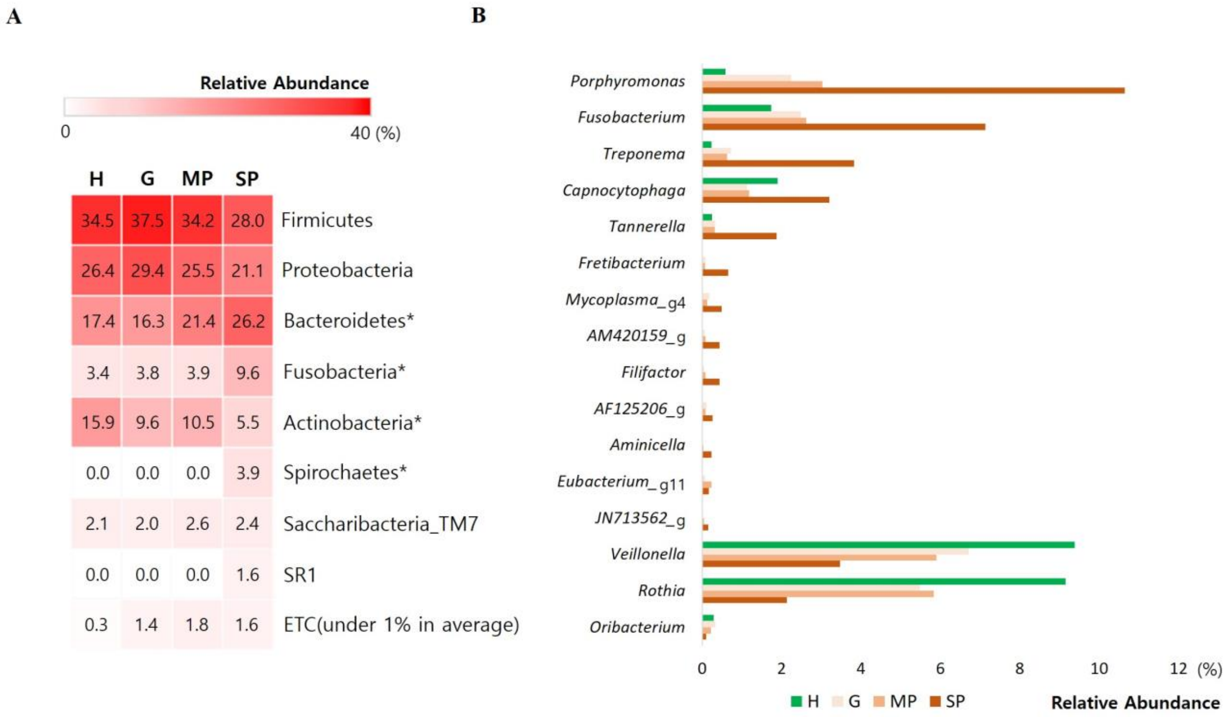

C

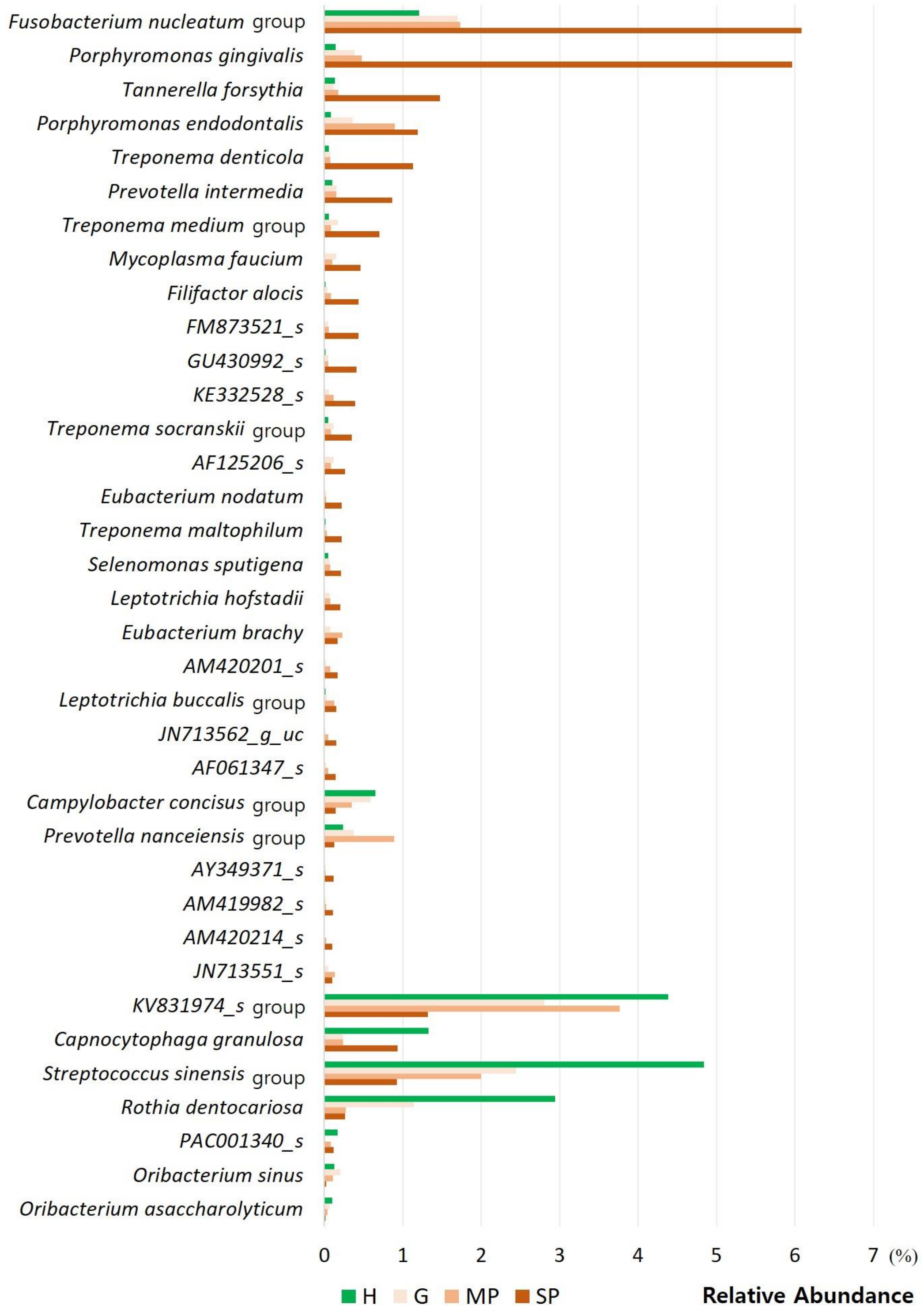
Fusobacterium nucleatum group
Porphyromonas gingivalis
Tannerella forsythia
Porphyromonas endodontalis
Treponema denticola
Prevotella intermedia
Treponema medium group
Mycoplasma faucium
Filifactor alocis
FM873521_s
GU430992_s
KE332528_s
Treponema socranskii group
AF125206_s
Eubacterium nodatum
Treponema maltophilum
Selenomonas sputigena
Leptotrichia hofstadii
Eubacterium brachy
AM420201_s
Leptotrichia buccalis group
JN713562_g_uc
AF061347_s
Campylobacter concisus group
Prevotella nanceiensis group
AY349371_s
AM419982_s
AM420214_s
JN713551_s
KV831974_s group
Capnocytophaga granulosa
Streptococcus sinensis group
Rothia dentocariosa
PAC001340_s
Oribacterium sinus
Oribacterium asaccharolyticum
0 1 2 3 4 5 6 7 (%)
H G MP SP
Relative Abundance

Differences in taxa distribution at the phylum, genus, and species levels among four groups of non-smoker females. The 16S rRNA gene sequencing was analyzed for non-smoker females consisting of 6 periodontally healthy subjects (H group), 16 patients with gingivitis (G group), 9 patients with moderate periodontitis (MP group) and 12 patients with severe periodontitis (SP group). **A.** The distribution pattern of the top 8 phyla except phylum under 1% in average in each sample group. *$p < 0.05$ by Kruskal–Wallis H test among four groups **B.** Genera significantly different among four groups among genera with >0.1% relative abundance in saliva. As the severity of periodontal disease increased, genera more abundant are the top 13 of the graph whereas genera more abundant in the healthy control are the bottom 3. **C.** Species significantly different between the four groups among genera with >0.1% relative abundance in saliva. As the severity of periodontal disease increased, species more abundant are the top 29 of the graph whereas species more abundant in the healthy control are the bottom 7. $p < 0.05$ was considered statistically significant by Kruskal–Wallis H test. H; healthy group, G; gingivitis group, MP; moderate periodontitis group, SP; severe periodontitis group.

Supplementa1 4. Quantitative comparison among periodontal groups of specific bacterial species analyzed by qRT-PCR in nonsmokers (bacterial percentage %)

Variables	Healthy (n=8)	Gingivitis (n=16)	Moderate Periodontitis (n=17)	Severe Periodontitis (n=18)	*p*	*p*Reg
P. gingivalis	0.125±0.280	0.287±0.919	0.371±0.574	2.297±6.588	0.002	0.109
T. forsythia	0.008±0.013	0.014±0.033	0.037±0.068	0.075±0.092	0.009	0.004
T. denticola	0.012±0.020	0.014±0.037	0.054±0.084	0.033±0.058	0.054	0.208
P. intermedia	0.053±0.106	0.026±0.044	0.013±0.020	0.071±0.092	0.048	0.316
P. endodontalis	0.003±0.002	0.042±0.062	0.040±0.047	0.059±0.080	0.338	0.057
F. alocis	0.001±0.002	0.016±0.035	0.020±0.036	0.044±0.076	0.003	0.033
F. nucleatum	0.833±1.168	0.375±0.424	0.566±0.661	0.401±0.601	0.748	0.347
P. micra	0.344±0.335	0.497±0.464	1.805±3.019	0.545±0.712	0.182	0.546
R. dentocariosa	0.255±0.464	0.113±0.210	0.019±0.017	0.015±0.021	0.076	0.005

Supplementa1 5. Quantitative comparison between non-smoker and smoker of specific bacterial species analyzed by qRT-PCR in SP group (bacterial percentage %)

Variables	Non-smoker (n=18)	Smoker (n=11)	*p*
P. gingivalis	2.297±6.588	1.530±2.317	0.857
T. forsythia	0.075±0.092	0.059±0.053	0.946
T. denticola	0.033±0.058	0.056±0.081	0.982
P. intermedia	0.071±0.092	0.082±0.128	0.500
P. endodontalis	0.059±0.080	0.089±0.146	0.753
F. alocis	0.044±0.076	0.082±0.122	0.787
F. nucleatum	0.401±0.601	0.597±0.788	0.893
P. micra	0.545±0.712	1.338±1.593	0.393
R. dentocariosa	0.015±0.021	0.073±0.139	0.345

Supplementa1 6. Quantitative comparison between male and female of specific bacterial species analyzed by qRT-PCR in MP group (bacterial percentage %)

Variables	Male (n=9)	Female (n=10)	*p*
P. gingivalis	0.420±0.726	0.264±0.352	0.327
T. forsythia	0.061±0.088	0.010±0.008	0.683
T. denticola	0.066±0.098	0.033±0.063	0.236
P. intermedia	0.018±0.025	0.008±0.010	0.683
P. endodontalis	0.042±0.058	0.034±0.033	0.935
F. alocis	0.027±0.050	0.012±0.007	0.288
F. nucleatum	0.667±0.730	0.406±0.543	0.369
P. micra	1.751±1.997	1.532±3.625	0.369
R. dentocariosa	0.016±0.023	0.021±0.009	0.041

Supplementa1 7. Quantitative comparison between male and female of specific bacterial species analyzed by qRT-PCR in SP group (bacterial percentage %)

Variables	Male (n=17)	Female (n=12)	*p*
P. gingivalis	1.268±2.031	3.051±8.018	0.565
T. forsythia	0.049±0.045	0.097±0.106	0.388
T. denticola	0.050±0.075	0.030±0.057	0.825
P. intermedia	0.062±0.108	0.094±0.102	0.376
P. endodontalis	0.071±0.121	0.071±0.092	0.626
F. alocis	0.069±0.101	0.042±0.089	0.565
F. nucleatum	0.476±0.653	0.474±0.726	0.825
P. micra	1.049±1.342	0.559±0.842	0.288
R. dentocariosa	0.056±0.114	0.011±0.014	0.215
